# Low Grip Strength Associated with Clinical Outcomes after Total Hip Arthroplasty ‐ A Prospective Case–Control Study

**DOI:** 10.1111/os.13007

**Published:** 2021-05-25

**Authors:** Gong Long, Chen Chao, Tan Ming‐sheng, Yi Ping

**Affiliations:** ^1^ Department of Orthopaedic China‐Japan Friendship Hospital Beijing China; ^2^ Peking Union Medical College, Chinese Academy of Medical College Beijing China; ^3^ Beijing Hospital of Traditional Chinese Medicine, Capital Medical University Beijing China

**Keywords:** Grip strength, Total hip arthroplasty (THA), Recovery, Muscle weakness, Bad results

## Abstract

**Objective:**

To assess whether low grip strength (GS) is associated with clinical outcomes after total hip arthroplasty (THA).

**Methods:**

A prospective case–control study was designed to assess 231 cases of primary THA between January 1, 2015 to May 1, 2018, at an urban tertiary care hospital. Patients were placed into two cohorts based on preoperative GS levels. Low GS in the present study was defined as GS lower than 26 kg for men and 16 kg for women in the dominant hand. Baseline data were prospectively collected and included patient demographics (age, sex, body mass index [BMI]), the surgeon's diagnoses, medical history, length of stay, and American Society of Anaesthesiologists' (ASA) score. Clinical outcomes included surgery‐ and prosthesis‐related variables. The Harris hip score (HHS) and the Short Form Health Survey (SF‐12) were completed at the baseline visit and at 1 and 2 years postoperatively in the outpatient department to assess the hip's function and quality of life. Differences in baseline data, length of study (LOS), 90‐day postoperative complications, and hospital readmissions were compared. Besides, the correlations between GS and Harris hip score (HHS) and Short Form score (SF‐12) were tested.

**Results:**

A total of 202 participants have completed records for analysis finally. The patients were followed up for an average of 24.8 months postoperatively (24–26 months). Eighty‐two patients (40.6%) had low GS before THA. Patients with low GS were more likely to be female, older, fracture of femoral head or neck as the primary cause, albumin <3.5 g/dL, and have a lower BMI, higher ASA score, increased rates of the pressure sore, blood transfusion, and LOS compared to normal GS (all *P* < 0.05). Also, patients in the low GS cohort showed a statistically significant increased unplanned hospital readmissions and decreased discharge home compared to normal GS (both *P* < 0.05). There was an increasing rate of complications between the two cohorts, for cardiac complications, pressure sore after THA, respiratory complications, urinary tract infection, stroke, and DVT (all *P* < 0.05). A partial correlation test by controlling medical comorbidities and demographic factors was used to determine the correlation between GS and HHS. There was a significant correlation between them (*r* = −0.673; *P* = 0.002). A similar condition was detected in the correlation between GS and SF‐12 (*r* = 0.645; *P* = 0.001).

**Conclusions:**

Clinicians should be encouraged to include GS assessment in their evaluation of patients who planned to undergo THA in order to optimize the treatment of high‐risk individuals.

## Graphical abstract

GS can serve as a useful indicator for assessing muscle weakness before primary THA. Clinicians should be encouraged to include GS assessment in their evaluation of patients who planned to undergo THA in order to optimize the treatment of high‐risk individuals.

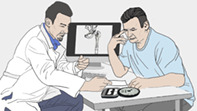

## Introduction

Total hip arthroplasty (THA) can reconstruct hip function to significantly improve the quality of life (QoL)^1^, ^2^. More than 230,000 patients in the United States alone and about 500,000 patients worldwide accept THA every year^3^. These figures increase by about 10% per year^4^, ^5^. Following THA, the majority of patients experience reductions in pain, improvements in function, and better health‐related quality of life^3^. However, not all patients achieve the same level of functional improvement after THA. Some patients who undergo THA continue to experience pain, limitations of activities of daily living, or limited range of motion even when no specific prosthesis‐related technical problem or failure mode can be identified, and mechanical or biologic problems have been ruled out^6^. Specifically, more than 30% of patients undergoing THA report moderate‐to‐severe activity limitations 2 years post‐THA^3^, ^4^. It is unclear which factors are associated with these limitations in function. Therefore, it is of great interest in orthopedic surgery to identify predictors for a good outcome. Surgeons keep exploring potential markers to predict such unsatisfactory results to help take preventive measures. This might facilitate handling the indication of THA with high responsibility and choosing the appropriate patients for surgery, especially in times of limited resources.

Reduced muscle strength is a risk factor for unsatisfactory results after THA. It makes it more difficult to regain lost balance and decreases the mechanical loading of the skeleton leading to reduced adaptive bone remodeling^6^, ^7^. To be specific, the muscle performance of trunk and lower extremities, such as quadriceps femoris, gluteus medius, and iliopsoas, determine the range of motion (ROM) of the hips and knees and patients' quality of life (QoL). Therefore, generalized loss of muscle strength and muscle mass thus leads to impaired neuromuscular function and decreased mechanical loading, consequently increasing the risk for both falls and fractures^7^. This condition threatens satisfactory results after THA.

Grip strength (GS) is a noninvasive marker of overall muscle strength and function and is recommended as a simple and valid assessment in the clinical setting^7^. Of note, clinicians and practitioners have realized the assessment of grip strength serves as an effective indicator of overall strength as there is numerous reliable evidence that GS might reflect the overall strength of an individual and give a good indication of overall strength and general heathy status^7^, ^8^. What is more, in spite of GS, which fails to directly reflect the performance of functional activities such as active ROM of knees and hips in the lower limbs and gait, this measure does distinguish the patients, especially the older adults, who have worse mobility and health status from other patients. Previous studies have revealed that low GS is associated with poor nutrition, low levels of fitness, and a range of adverse outcomes, including limited functional activities, disability, prolonged length of stay (LOS) in hospitalized patients, and even mortality^8^, ^9^, ^10^. Also, in both cross‐sectional and longitudinal studies, low GS has also been seen among sarcopenia, loss of skeletal muscle mass, and aging. Reduced muscular strength, which can be measured by GS, has been consistently linked with early death, disability, and illness. A previous systematic review by Denk *et al*. demonstrated that all the 11 studies included confirmed a correlation between decreased GS and the risk of hip fractures^11^. Li *et al*. showed that the patients with lower 25OHD levels had significantly lower BMD of the femoral neck. The patients with lower handgrip strength had significantly lower BMD of the lumbar spine, femoral neck, and total hip^12^. Another study has revealed that the assessment of GS, along with bone mineral density (BMD), was correlated with a rising risk of fragility fractures^13^. Although the direct cause of hip fractures is falling and other trauma, scholars assumed that GS might predict the risk of falls. In these regards, low GS may be relevant to the recovery from THA.

Accurate prediction of whether patients will be able to have good recovery from THA may allow them to set appropriate goals for their postoperative rehabilitation and is critical for the planning of effective rehabilitation interventions. From our perspective, the development of standardized and valid preoperative assessment methods that may predict GS would be useful for comprehensive rehabilitation after THA. However, until now, to our best knowledge, information on the prognostic value of GS has been limited in the orthopedic field and mainly obtained from internal medicine. Little information about GS's assessment before THA and whether it could help predict the patients' prognosis is worthy of being investigated. Therefore, the purpose of this study is as follows: (i) to evaluate the prevalence of low GS among patients undergoing THA; (ii) to assess whether low GS is predictive for the bad results after THA and (iii) to discuss the superiority in the application of this simple indicator. To our knowledge, this is the first study to evaluate the predictive role of GS in the clinical outcomes after THA. We hypothesized that GS could distinguish the patients who had unsatisfactory results after THA from others.

## Methods and Materials

### Participants

From January 1, 2015 to May 1, 2018, there were 231 patients who underwent primary THA included in the study. This is a prospective study. This study was approved by the human research ethics committee of the authors' affiliated institutions, and all participants provided us with written informed consent.

Inclusion criteria for the case group included (i) undergoing primary and unilateral THA; (ii)had a completed data including clinical evaluation and examination used for comparison; and (iii) a prospective study.

Participants were excluded due to: (i)loss of follow‐up/ incomplete data (*n* = 4); (ii) inadequate ability to perform the tests due to muscular atrophy of upper extremity alone on the dominant side (*n* = 6); (iii) vocation/recent (<6 months) exercise which could merely affect the muscle strength of upper extremity (such as pull‐up hobbyist) before THA (*n* = 2); (iv) cancer history (*n* = 8) and (v) comorbidities which are considered to produce a significant influence on patients' outcome were also excluded, including *severe* diabetes, *poor* renal function, *severe* hypertension, ischemic heart disease, liver cirrhosis or other diseases (*n* = 9).

### Surgery

The patients were performed with general anesthesia or combined spinal and epidural anesthesia in a lateral position. THA was performed using a standard Watson‐Jones approach. Following adequate exposure of the acetabulum, the capsule was resected to reach the true acetabulum. Generally beginning with 32 mm reamers, the acetabulum was reamed until it reached the medial wall of the true acetabulum. Then, the acetabulum was reconstructed with an acetabular prosthesis (Waldemar Link, Munich, Germany). After the acetabular prosthesis was implanted, the proximal femur was reamed with straight reamers. The proximal transverse osteotomy was done under the lesser trochanter using a reciprocating saw before the appropriate stem size was obtained through sequential rasping. A fully coated tapered stem (LCU Hip system, Waldemar Link, Munich, Germany) was inserted. Both the acetabular cup and femoral stem were cementless. All patients were routinely given prophylactic cefuroxime 1 h preoperatively and the first 24 h postoperatively (1.5 g, iv, tid). Preventive anticoagulant therapy [10 mg rivaroxaban every day or 2850 international units (IU) low‐molecular‐weight heparin (LMWH) (body weight < 90 kg) or 5700 IU (bodyweight >90 kg)] began within 12 h postoperatively and continued for 28 days at least. Patients were encouraged to begin weight‐bearing as soon as tolerable with the help of ambulatory aids (usually within the first 24 h). They then were allowed to discontinue the assistance of aids as they could ambulate without a limp (usually within 6–12 weeks). The physical therapist performed a functional exercise for all patients. The postoperative X‐ray alignment on standard views was evaluated by two experienced orthopaedists, and all enrolled cases have a good quality of component placement without the femoral stems placed in a varus or valgus position. Patients were kept their anatomical abductor lever arm and offset after surgery.

### Data Collection and Assessments

#### Baseline Data

Baseline data were prospectively collected and included patient demographics (age, sex, body mass index [BMI]), surgeon's diagnoses, medical history, and length of stay.

#### Grip Strength Testing

Participants were tested at least three times using a grip strength dynamometer (Jk‐w, Jingkaida, Beijing, China) several days prior to operation. The standard position for testing grip strength is the supine position with the upper limb relaxed on the bed. The elbow is extended without any flexion. Low GS in the present study was defined as GS lower than 26 kg for men and 16 kg for women in the dominant hand, according to the references of the FNIH Sarcopenia Project^14^. According to the criteria, the patients were classified into low GS and normal GS cohort and compared.

#### American Society of Anaesthesiologists (ASA) Score

The American Society of Anesthesiologists (ASA) score is used for evaluating the patient's physical fitness for procedures. It has also been analyzed for the risk prediction of mortality and complications since the score is simple, reliable, readily available, focusing only on the status of patients at the time of admission^15^. The higher score represents an increased risk for mortality and complications after THA in the present study.

#### Harris Hip Score (HHS)

The Harris Hip Score (HHS) is a doctor‐reported measure that involving the domains for pain severity (1 item, 0–44 points), function (7 items, 0–47 points), absence of deformity (1 item, 0–4 points), and range of motion (2 items, 0–5 points). Scores range from 0 (worse disability) to 100 (less disability)^16^. A high score means a better hip function. This measure was completed at the baseline visit and at 1 and 2 years postoperatively in the outpatient department to assess the hip's function of patients.

#### Short Form Health Survey (SF‐12)

Short Form Health Survey (SF‐12) is a worldwide used, multipurpose, short‐form generic evaluation for an individual's health status, including physical and mental health status, respectively^17^. A higher score is considered a better health status. This measure was completed at the baseline visit and at 1 and 2 years postoperatively in the outpatient department to assess the QoL of the patients.

#### Complications

Complications that occurred 90 days postoperatively were recorded. A major complication was defined as myocardial infarction, postoperative mortality, sepsis, stroke, PJI, and death. Cardiac diseases were identified as arrhythmia, myocardial infarction, cardiac failure, coronary artery disease, and cardiomyopathy. Deep vein thrombosis (DVT) was confirmed by angiography and color ultrasound. More postoperative complications revealed an increased incidence of reoperation and more costs for individuals and medical insurance.

### Sample Size

Using G Power 3.1.9.2, posthoc achieved study power was calculated for the effect size of 0.3, error of the first type 0.05, and the total number of respondents with the number of 202 patients undergoing THA. The calculated study power equals 92.13%, which indicates good study power.

### Statistical Analysis

Countable variables were presented as percentages and compared using the chi‐square test. Normally distributed continuous and non‐normally distributed continuous data were respectively presented as mean ± SD and the median and range. A *t*‐test was used to compare clinical outcomes between groups. A multivariate logistic regression analysis was then performed to estimate the odds ratio (*OR*) and the associated 95% confidence interval (*CI*) to identify factors independently associated with 90‐day major complications. To control for confounding variables, we used a multivariate logistic regression analysis to assess associated risk factors for 90‐day major complications. Patient demographic factors and preoperative status were controlled for multivariate analysis. The Pearson correlation coefficient (coefficient, *r*) was used to test the correlation between preoperative GS and HHS and SF‐12 improvement by controlling parameters with a correlation value of more than 0.5. *P* < 0.05 was considered statistically significant, and power analysis was ≤0.9. All data were collected and analyzed using SPSS for Windows version 18.0 (SPSS Inc., Chicago, IL).

## Results

### General Results

A total of 202 participants have completed records for analysis finally. The patients were followed up for an average of 24.8 months postoperatively (24–26 months). Of this total, 82 participants (40.6%) had low GS.

### Comparisons between Patients with Low GS and Normal GS

As illustrated in Table 1, compared to normal GS, patients with low GS were more likely to be older (61.2 ± 5.3 *vs* 65.2 ± 6.2, *P* < 0.001), female (46.7% *vs* 63.4%, *P* = 0.028), fracture of femoral head or neck as the primary cause (18.3% *vs* 41.5%, *P* = 0.008), albumin <3.5 g/dL (6.7% *vs* 18.3%, *P* = 0.020), and have a lower BMI (25.2 ± 4.2 *vs* 23.4 ± 3.2, *P* = 0.001), higher ASA score (47.5% *vs* 63.4%, *P* = 0.008), increased rates of pressure sore before surgery (2.5% *vs* 8.5%, *P* = 0.034), blood transfusion (12.5% *vs* 24.4%, *P* = 0.045), and LOS (7.2 ± 3.8 *vs* 12.2 ± 3.1, *P* < 0.001).

**TABLE 1:** Comparisons of demographic and THA‐related operative data for patients with low GS and normal GS

Variable	Low GS (*n* = 82)	Normal GS (*n* = 120)	*P* value
Mean age (years) (SD)	65.2 ± 6.2	61.2 ± 5.3	<0.001^*^
Female (%)	52 (63.4)	56 (46.7)	0.028^*^
Mean BMI (kg/m2) (SD)	23.4 ± 3.2	25.2 ± 4.2	0.001^*^
Smoking history (%)	21 (25.6)	33 (27.5)	0.892
Heavy drinking (%)	10 (12.2)	14 (11.7)	0.915
Pressure sore before THA (%)	7 (8.5)	3 (2.5)	0.034^*^
Hypertension requiring medication (%)	26 (31.7)	35 (29.1)	0.818
Cardiac diseases (%)	16 (19.5)	18 (15.0)	0.516
COPD (%)	6 (7.3)	8 (6.7)	0.918
Dialysis (%)	2 (2.4)	2 (1.7)	0.899
Diabetes mellitus (%)	25 (30.5)	34 (28.3)	0.863
Preoperative albumin <3.5 g/dL (%)	15 (18.3)	8 (6.7)	0.020^*^
General anesthesia (%)	14 (17.1)	18 (15.0)	0.841
Diagnose			
AVNFH	27 (32.9)	64 (53.3)	0.008^*^
Fracture of femoral head or neck	34 (41.5)	22 (18.3)	
DDH	12 (14.6)	14 (11.7)	
RA	3 (3.7)	6 (5.0)	
Posttraumatic arthritis	3 (3.7)	5 (4.2)	
Others	3 (3.7)	9 (7.5)	
ASA Class			
I‐II	30 (36.6)	63 (52.5)	0.047^*^
III‐IV	51 (62.2)	57 (47.5)	
V‐VI	1 (1.2)	0 (0)	
Mean operative time (minutes) (SD)	61.5 ± 8.2	62.7 ± 7.8	0.294
Perioperative blood transfusion rate (%)	20 (24.4)	15 (12.5)	0.045^*^
Length of stay (days)	12.2 ± 3.1	7.2 ± 3.8	<0.001^*^

ASA, American Society of Anaesthesiologists; AVNFH, avascular necrosis of femoral head; BMI, Body Mass Index; COPD, chronic obstructive pulmonary disease; DDH, developmental dysplasia of the hip; GS, grip strength; RA, rheumatoid arthritis; SD, standard deviation; THA, total hip arthroplasty.

^*^ Indicates statistical significance.

As illustrated in Table 2, patients in the low GS cohort showed a statistically significant increased unplanned hospital readmissions (15.9% *vs* 4.2%, *P* = 0.002) and decreased discharge home (95.8% *vs* 83%, *P* = 0.006) compared to normal GS. In addition, there was an increasing rate of complications between the two cohorts, for pressure sore after THA (7.3% *vs* 1.7%, *P* = 0.031), DVT (11.0% *vs* 2.5%, *P* = 0.028), cardiac complications (11.0% *vs* 2.5%, *P* = 0.028), stroke (9.8% *vs* 1.7%, *P* = 0.023), respiratory complications (7.3% *vs* 1.7%, *P* = 0.098), and urinary tract infection (9.8% *vs* 1.7%, *P* = 0.023).

**TABLE 2:** Comparisons of adverse outcomes for patients with low GS and normal GS

Variable	Low GS (*n* = 82)	Normal GS (*n* = 120)	*P* value
Unplanned hospital readmissions (%)	13 (15.9)	5 (4.2)	0.002^*^
Pressure sore after THA (%)	6 (7.3)	2 (1.7)	0.031^*^
Dislocation after THA (%)	5 (6.1)	1 (0.8)	0.082
Wound dehiscence (%)	3 (3.7)	3 (2.5)	0.957
Wound infection (%)	2 (2.4)	3 (2.5)	0.665
PJI (%)	1 (1.2)	1 (0.8)	0.652
DVT (%)	9 (11.0)	3 (2.5)	0.028^*^
Cardiac complications (%)	9 (11.0)	3 (2.5)	0.028^*^
Stroke (%)	8 (9.8)	2 (1.7)	0.023^*^
Respiratory complications (%)	6 (7.3)	2 (1.7)	0.098^*^
Renal complications (%)	1 (1.2)	1 (0.8)	0.652
Urinary tract infection (%)	8 (9.8)	2 (1.7)	0.023^*^
Sepsis (%)	1 (1.2)	0 (0.0)	0.848
Death (%)	2 (2.4)	0 (0.0)	0.319
Discharge disposition (%)			
Home	68 (83.0)	115 (95.8)	0.006^*^
Skilled nursing or rehabilitation	12 (14.6)	5 (4.2)	
Death	2 (2.4)	0 (0.0)	

DVT, deep vein thrombosis; GS, grip strength; PJI, periprosthetic joint infections; THA, total hip arthroplasty.

^*^ Indicates statistical significance.

### Risk Factors for Adverse Outcomes Following the THA

As illustrated in Table 3, controlling for age, sex, and BMI, the patients having low GS were more likely to experience a major complication within 90 days of surgery (*OR* = 7.11; 95% *CI*: 7.01–7.19; *P* < 0.001). Other risk factors included smoking history (*OR* = 2.12; 95% *CI*: 2.00–2.23; *P* = 0.012), pressure sore before THA (*OR* = 3.42; 95% *CI*: 3.25–3.60; *P* = 0.023), preoperative albumin <3.5 g/dL (*OR* = 3.52; 95% *CI*: 3.34–3.70; *P* = 0.021), and ASA 4 or greater (*OR* = 4.02; 95% *CI*: 3.82–4.22; *P <* 0.001) (Table 3).

**TABLE 3:** Adjusted risks of 90‐day postoperative major complications after THA

Risk factor	*OR*	95% *CI* ^†^	*P*‐value
Low GS	7.11	7.01–7.19	<0.001^*^
Smoking history	2.12	2.00–2.23	0.012^*^
Heavy drinking	1.64	1.56–1.72	0.453
Pressure sore before THA	3.42	3.25–3.60	0.023^*^
Hypertension requiring medication	1.85	1.76–1.94	0.253
Cardiac diseases	2.45	2.33–2.57	0.321
COPD	2.31	2.19–2.43	0.762
Dialysis	1.78	1.69–1.87	0.213
Diabetes mellitus	2.21	2.10–2.32	0.543
Preoperative albumin <3.5 g/dL	3.52	3.34–3.70	0.021^*^
General anesthesia	2.11	2.00–2.22	0.212
ASA 4 or greater	4.02	3.82–4.22	<0.001^*^
Perioperative blood transfusion rate	2.52	2.39–2.65	0.762

Odds ratios, as well as 95% CIs, were shown.

ASA, American Society of Anaesthesiologists; BMI, body mass index; CI, confidence interval; COPD, chronic obstructive pulmonary disease; GS, grip strength; OR, odds ratio; THA, total hip arthroplasty.

^*^ Indicates statistical significance.

^†^ Fully adjusted by confounding variables.

### Correlation Analysis between GS Level and Patient‐Reported Outcomes for THA

A partial correlation test by controlling medical comorbidities and demographic factors was used to determine the correlation between GS and HHS. There was a significant correlation between them (*r* = −0.673; *P* = 0.002).

A partial correlation test by controlling medical comorbidities and demographic factors showed a significant correlation between GS and SF‐12 (*r* = 0.645; *P* = 0.001).

## Discussion

### Main Findings

Low GS (<26 kg for men and <16 kg for women in the dominant hand) is associated with poor nutritional status, low levels of fitness, and a range of adverse outcomes, including limited functional activities, disability, prolonged LOS, as well as even mortality^8^, ^9^, ^10^. The extent of these effects on patient‐reported outcome measure (PROM), postoperative complications, and unplanned readmissions following primary THA is unclear. In this study, through conducting a prospective case–control design, we concluded that low GS before THA is a significant risk factor for increased LOS, unplanned hospital readmissions, worse PROM, and postoperative complications.

### Predictive Role of Low GS for the Clinical Outcomes Following Orthopedic Surgery

A number of previous studies have examined the predictive role of low GS for the clinical outcomes following orthopedic surgery^18^, ^19^, ^20^, ^21^, ^22^. For instance, Shen *et al*. reported patients with high preoperative GS to display a better surgical outcome in terms of disability and health status 6 months after spine surgery^18^. Selakovic *et al*. showed multivariate regression analysis adjusted for age and gender revealed that grip weakness was an independent predictor of worse functional outcome at 3 and 6 months after hip fracture for both genders and in all age populations^19^. Similarly, Hershkovitz *et al*. suggested that grip strength is independently associated with rehabilitation outcomes in post‐acute frail hip fracture patients^20^. A few studies have also investigated the predictive role of low GS in arthroplasty patients. Hashimoto *et al*. have investigated the effects of preoperative GS on stair ascent and descent ability in patients undergoing total knee arthroplasty^21^. In another study, Shyam *et al*. examined this simple GS test may be highly beneficial preoperatively in identifying those patients likely to require longer inpatient stays and, therefore, those who would benefit from early nutritional intervention and focused physiotherapy on a cohort of total hip and total knee arthroplasty patients^22^. Similar to our study, they chose to measure the GS in patients who planned to undergo THA in the pre‐admission clinic and reported similar findings to our study about the LOS, with an average delay in‐hospital stay of 5 days in the low GS cohort compared to the normal GS (12.2 ± 3.1 *vs* 7.2 ± 3.8). However, unlike in our study, Hashimoto *et al*. and Shyam *et al*. did not investigate whether there existed a statistically significant difference in PROM and postoperative complications between the two cohorts.

### Analysis of the Predictive Role of Low GS for the Clinical Outcomes Following THA

Our study further corroborated previous findings^19^, ^23^, ^24^, ^25^, ^26^ about the patient characteristics associated with low GS in this population undergoing primary THA. As expected, there were significant differences in preoperative characteristics between the low GS and normal GS cohorts. Patients with low GS were more likely to be female, older, have a lower BMI and higher ASA score. Also, these patients were more likely to have increased rates of pressure sore before THA, preoperative albumin < 3.5 g/dL, and perioperative blood transfusion rate. This meant patients with low GS experienced worse nutritional status and limitations in mobility. Multivariate analysis was utilized to demonstrate that even when accounting for these differences in preoperative characteristics, low GS patients remained at risk for 90‐day postoperative major complications after THA. The statistically significant variables in the univariate analysis in Table 1 were treated as potential confounders and analyzed in the multivariate analysis.

Low GS was present in 31.9% in men and 48.1% in women of the total primary THA patients in this study. This percentage is higher than 5% in men and 18% in women aged 65 years and above in multiple populations (Europe/North America, South America, Middle East, Africa, South East Asia, South Asia, and China)^27^, and 18.0% and 38.4% in Australian men and women, respectively^28^. The reason for the difference in reported prevalence about low GS could be related to the race and the selection of the studied population. Our population, which is based on the patients undergoing THA in Asia, may have worse overall muscle strength and function compared to the general elderly population. When considering such a considerable percentage, these equate to significant effects on the over 600,000 primary THAs performed annually^25^. Low GS patients were more likely to have a worse functional outcome and quality of life reflected by HHS and SF‐12 scores. Besides, they were nearly seven times (*OR*, 7.11) as likely to had a higher risk of experiencing a major complication within 90 days of surgery, which in this study included myocardial infarction, postoperative mortality, sepsis, stroke, PJI, and death, after controlling for confounding variables. Limitations in mobility, reflected by the GS, may explain the high risk for these complications and other adverse outcomes, including pressure sore after THA, respiratory complications, urinary tract infection, and DVT. Of note, despite no statistically significant difference in dislocation after THA between two cohorts, the percentage in the low GS cohort is nearly 3.6 times that in the normal GS cohort (6.1 *vs* 1.7, *P* = 0.082). This could mean GS has a close association with the function of the abductor muscle group.

### Clinical Implications

Our findings have certain implications. First, there is an ongoing urgent need to identify THA patients at increased risk for worse outcomes. Identifying this population of patients allows for the initiation of prevention and specific intervention to avoid the debilitating consequences of THA. Especially for patients undergoing THA due to hip fracture, other methods measuring muscle strength such as gait speed and muscle mass cannot be assessed before surgery. Instead, GS measurement is simple enough to be applied in the clinical setting. Therefore, confirmation of the prognostic value of HGS assessed in the THA setting is very significant. To the best of our knowledge, this is the first study to evaluate the role of HGS in predicting the clinical outcomes following THA for both gender and all ages. Second, muscle weakness, reflected by GS, is a modifiable risk factor that can be improved. It has been well known that strengthening exercises, especially for patients undergoing a selective operation, had positive effects on various outcomes after THA^25^. Therefore, GS measurement before THA could be considered in the design for individualized treatment plans to improve functional recovery.

### Limitation

Our study has several limitations. First, the sample size was relatively small. Patients were collected only from one single center. Second, analyzed complications in this study are limited to medical complications 90 days postoperatively. Therefore, we do not have data on delayed complications or orthopedic‐specific perioperative complications. Third, despite being discussed, there are significant differences in preoperative characteristics between the cohorts. The multivariate analysis attempts to account for these differences; however, there remains a risk that the analysis missed important confounding factors not included in the model. Therefore, the potential confounding effects of these factors on grip strength could not be determined. Lastly, we are also completely aware of the fact that our GS measurement method is different from the standardized approach advocated by the American Society of Hand Therapists (ASHT), where the subjects are tested in a seated position^26^. GS was measured before THA in the supine position since we noticed that it is unrealistic for a portion of patients, such as those with hip fractures, to complete the GS test in a seated position. Measuring GS after THA in a seated position would definitely be a more standardized way to assess muscle strength. However, we have standardized the way to measure GS in all the included patients, and this approach was also used by other authors^19^, ^27^, ^28^. Consequently, it is reasonable to assume that measuring GS in the supine position is an appropriate way to assess function.

### Conclusion

GS can serve as a useful indicator for assessing muscle weakness before primary THA. In this prospective case–control study, we identified low GS as a modifiable significant risk factor for increased LOS, 90‐day postoperative complications, worse functional results, and hospital readmissions. This study further supports the need for screening and preoperative intervention for patients at this risk group. Clinicians should be encouraged to include GS assessment in their evaluation of patients who planned to undergo THA in order to optimize the treatment of high‐risk individuals.
